# Identification of spleen tyrosine kinase as a potential therapeutic target for esophageal squamous cell carcinoma using reverse phase protein arrays

**DOI:** 10.18632/oncotarget.24853

**Published:** 2018-01-01

**Authors:** Mustafa A. Barbhuiya, Manoj K. Kashyap, Vinuth N. Puttamallesh, Rekha Vijay Kumar, Xinyan Wu, Akhilesh Pandey, Harsha Gowda

**Affiliations:** ^1^ McKusick-Nathans Institute of Genetic Medicine, Johns Hopkins University School of Medicine, Baltimore, MD, USA; ^2^ Sidney Kimmel Comprehensive Cancer Centre, Johns Hopkins University School of Medicine, Baltimore, MD, USA; ^3^ School of Life and Allied Health Sciences, Glocal University, Saharanpur, India; ^4^ Institute of Bioinformatics, International Technology Park, Bangalore, India; ^5^ Amrita School of Biotechnology, Amrita Vishwa Vidyapeetham, Kollam, India; ^6^ Department of Pathology, Kidwai Memorial Institute of Oncology, Bangalore, India; ^7^ Department of Biological Chemistry, Johns Hopkins University School of Medicine, Baltimore, Maryland, USA; ^8^ Department of Oncology, Johns Hopkins University School of Medicine, Baltimore, Maryland, USA; ^9^ Department of Pathology, Johns Hopkins University School of Medicine, Baltimore, Maryland, USA; ^10^ Manipal Academy of Higher Education (MAHE), Manipal, India

**Keywords:** ESCC, SYK, entospletinib, RPPA

## Abstract

The vast majority of esophageal cancers in China, India and Iran are esophageal squamous cell carcinomas (ESCC). A timely diagnosis provides surgical removal as the main therapeutic option for patients with ESCC. Currently, there are no targeted therapies available for ESCC. We carried out reverse phase protein array-based protein expression profiling of seven ESCC-derivedcell lines and a non-neoplastic esophageal epithelial cell line (Het-1A) to identify differentially expressed proteins in ESCC. SYK non-receptortyrosine kinase was overexpressed in six out of seven ESCC cell lines that were used in the study. We evaluated the role of SYK in ESCC using the pharmacological inhibitor entospletinib (GS-9973) and siRNA-based knock down studies. Entospletinib is a selective inhibitor of SYK, which is currently being evaluated in phase II clinical trials for hematological malignancies. Using *in vivo* subcutaneous tumor xenografts in mice, we demonstrate that treatment with entospletinib significantly inhibits tumor growth. Further clinical studies are needed to prove the efficacy of entospletinib as a targeted therapeutic agent for treating ESCC.

## INTRODUCTION

The cancers of esophagus are mainly of two types – esophageal adenocarcinoma (EAD) and esophageal squamous cell carcinoma (ESCC). Esophageal cancer is the eighth most commonly occurring cancer and the sixth leading cause of cancer deaths worldwide [1]. In contrast to EAD, ESCC is more common in developing countries including India, China, and Iran [1, 2]. The incidence of ESCC is more common in males, with the male: female ratio being 2:1. Approximately 83% of total incidence of ESCC and 86% of deaths occur in developing countries [2]. The five-year survival rate is only 10-17% [3]. The major risk factors associated with ESCC include tobacco, alcohol, red meat, hot drinks, poor oral hygiene, ingestion of mycotoxins, salted food, smoked foods, and deficiency of essential micronutrients such as vitamin A and zinc, genetics and conditions like Plummer-Vinson/Patterson-Kelly syndrome [4–7]. Genomics and proteomics approaches have previously been used to explore molecular alterations associated with ESCC [1, 4, 8, 9].

Reverse phase protein arrays (RPPAs) have emerged as a non-mass spectrometry based high-throughput platform to carry out proteomic profiling studies. They have been used to identify potential biomarkers using biofluids, cell and tissue lysates. In recent years, RPPA has been utilized for profiling breast cancers [10], endometrioid and clear cell carcinomas [11] head and neck squamous cell carcinoma (HNSCC) patient derived xenografts, [12] studying mTOR activation in sorafenib-resistant hepatocellular carcinoma (HCC) [13], for biomarker discovery [14], characterization of signaling pathways [15] and for distinguishing different cancers [16]. We used RPPA for profiling ESCC cell lines to identify differentially expressed proteins in ESCC. We further validated SYK as a potential therapeutic target using *in vitro* and *in vivo* models through genetic and pharmacological inhibition of the target.

## RESULTS

### Differentially expressed proteins in ESCC cell lines

**Figure 1:**
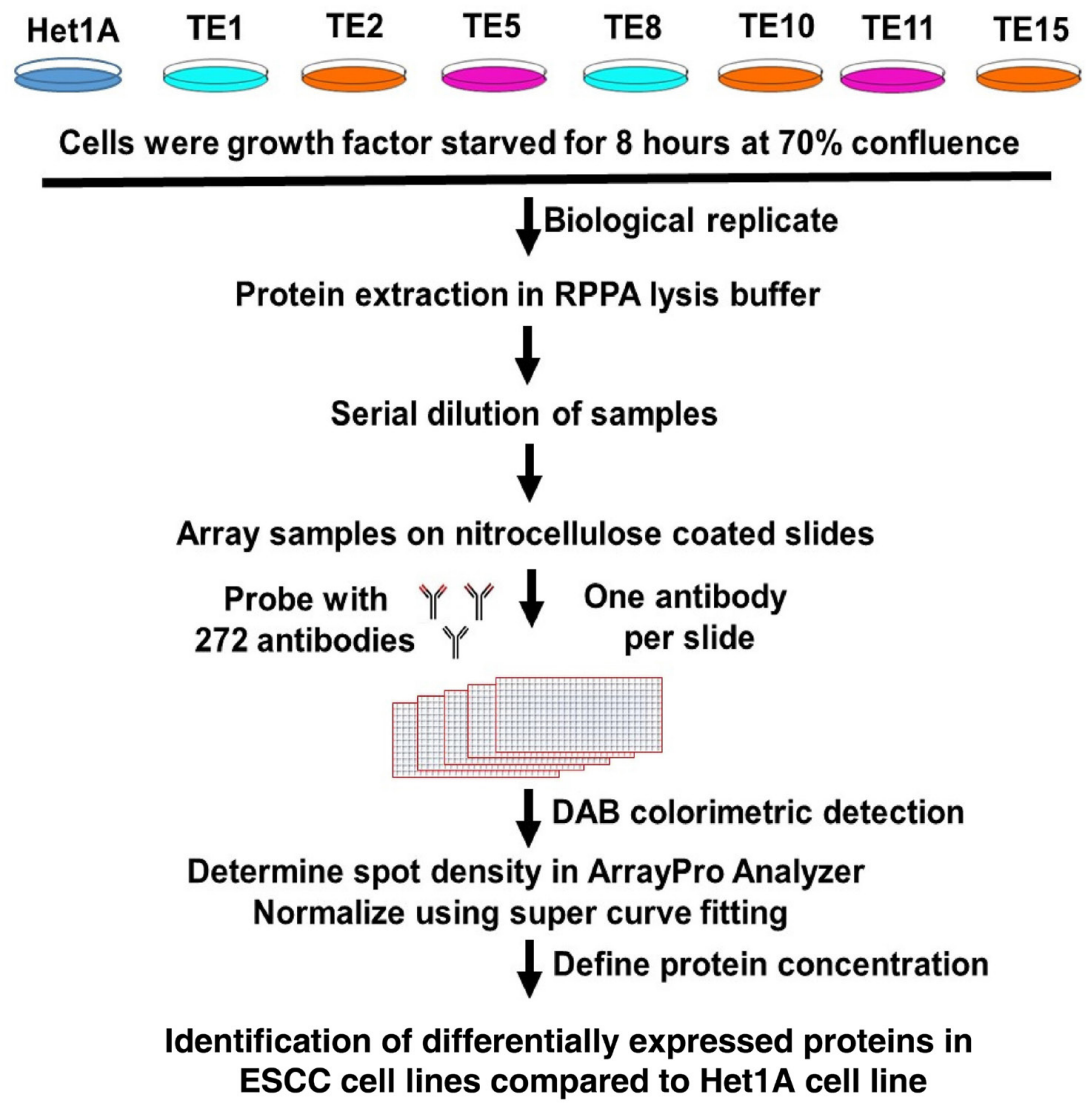
Experimental workflow followed for carrying out RPPA based protein expression profiling

**Figure 2:**
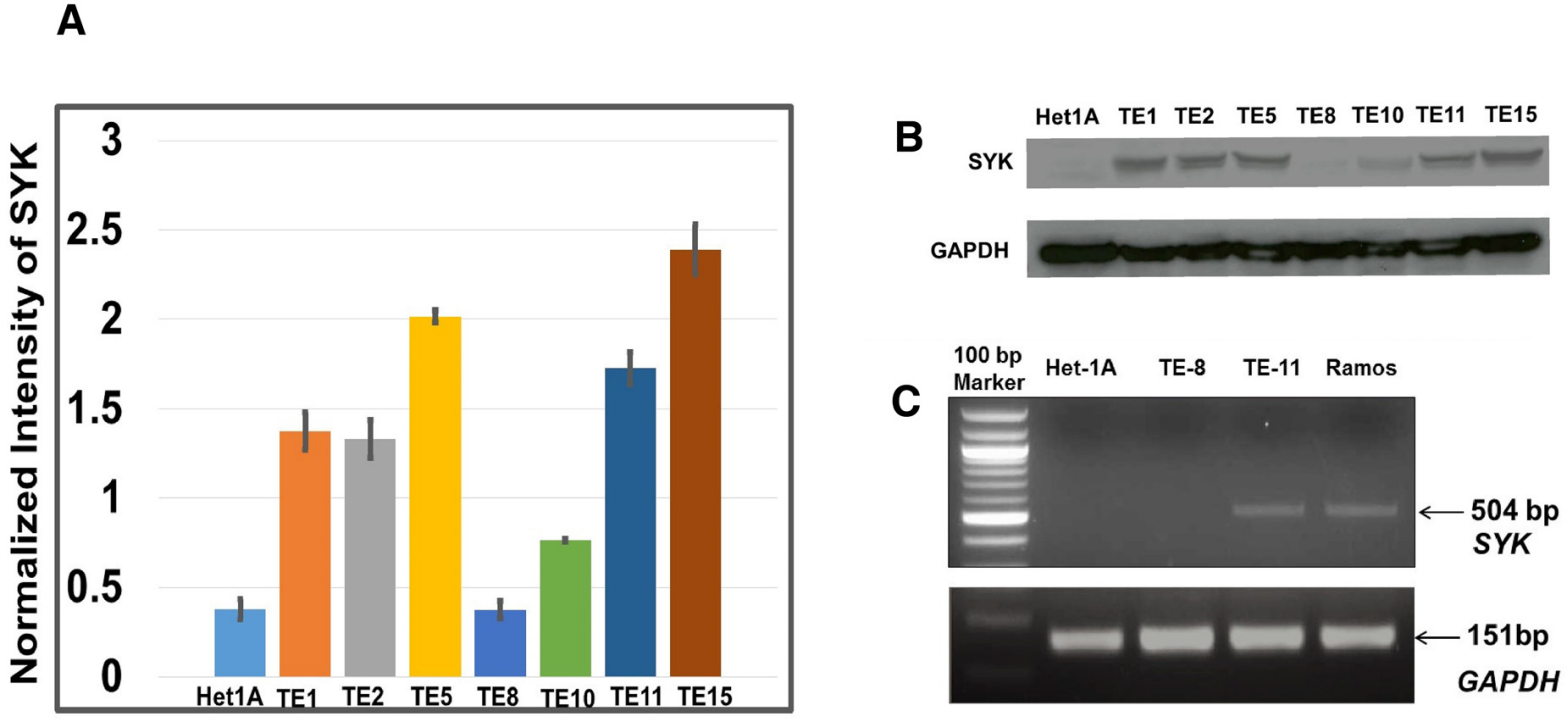
Expression of SYK across ESCC cell lines and non-neoplastic esophageal epithelial cell line **(A)** Histogram showing expression of SYK in ESCC cell lines as determined by RPPA array. **(B)** Expression of SYK across ESCC cell lines by Western blotting. **(C)** RT-PCR based evaluation of SYK expression in TE8, TE11 and Ramos cell lines. Ramos cell line was used as positive control.

Protein expression profiling of a panel of ESCC cell lines and Het-1A, a non-neoplastic esophageal epithelial cell line was carried out using RPPA (Figure 1). This led to identification of several proteins that were differentially expressed in ESCC cell lines as compared to Het-1A (Supplementary Table 1). These differentially expressed proteins included caveolin 1 (CAV1), cadherin 1 (CDH1), claudin 7 (CLDN7), cadherin 3 (CDH3), and PAR proteins, which were all 2-fold overexpressed in ESCC cell lines as compared to Het-1A cells (Supplementary Figure 1). SYK protein was overexpressed more than 3-fold in five of the seven cell lines (TE1, TE2, TE5, TE11 and TE15) (Figure 2A, 2B) and X-fold in the sixth cell line (you say overexpressed in 6 out of 7 cell lines!!!). CAV1, one of the three members of caveolin family, is a 21 kDa scaffoldingprotein that has been implicated in tumorigenesis and metastasis and involved in different cellular pathways including endocytosis, lipid homeostasis and signal transduction [17, 18]. In a number of cancer types including ovarian [19], colon [20] and breast cancer [21], CAV1 was found to be downregulated. In contrast, there are also number of malignancies where CAV1 was found to be overexpressed e.g. pancreatic adenocarcinoma [18, 22], prostate cancer [22, 23], non-small cell lung carcinoma, renal cell carcinoma (RCC) and glioblastoma [17]. In our study, we found that CAV1 was overexpressed in ESCC cell lines, which was in agreement with previous studies on CAV1 in ESCC [24–26].

Expression of claudins is known to be dysregulated in different cancers including HNSCC [27] breast cancer [28] ovarian [29] and gastric cancer [30]. In our study, we observed >2-fold change in CLDN7 expression in all the TE-series cell lines except TE8. The undetectable levels of CLDN7 in TE8 cell line is in agreement with a previous study on ESCC cell line where CLDN7 was found to be expressed in TE1, TE2, and TE11 but not in TE8 cell line [31]. CLDN7 shows difference in expression pattern between ESCC cell lines and surgically resected ESCC tissue samples. We and others observed expression of CLDN7 in TE-series ESCC cell lines, but expression of CLDN7 was found to be downregulated in ESCC tissue sections as compared with primary esophageal keratinocytes [31]. This study using 3-D cultures showed that the CLDN7 expression is dependent on the microenvironment of the tissue, thus observations of CLDN7 in the homogeneous population of TE cell lines may not reflect the outcome of tumor-microenvironment interaction [31]. Decrease in expression of CLDN7 protein has been reported in ESCC as compared to the prickle layer of the normal esophageal squamous epithelium [32].

### SYK is overexpressed in most ESCC cell lines

SYK was overexpressed >2-fold in 6 out of 7 ESCC cell lines as compared to normal esophageal epithelial cell line (Figure 2A, 2B). We used TE8 (SYK negative) and TE11 (SYK positive) lines for functional studies using siRNA based silencing of SYK. We screened for SYK expression by semi-quantitative reverse transcriptase PCR and observed high expression in TE11 cell line but could not detect expression in normal Het-1A and TE8 cells (Figure 2C). In recent years, there have been extensive studies focused on SYK, which is a non-receptor tyrosine kinase. SYK consists of a tyrosine kinase domain, two adjacent src homology (SH2) domains, but no SH3 domain [33]. SYK is a 72 kDa protein containing 635 amino acids and encoded by a gene located on chromosome 9q22 [33]. SYK is known to play a role in tumor invasion and progression and migration depending on the cell type [34–36]. SYK expression has been reported in B, T and NK cells and it is an integral part of BCR signaling of B cell precursors as well as mature B lymphocytes [37]. SYK is an upstream regulator of PI3K in BCR signaling pathway. SYK has the ability to activate PI3K by using adaptor proteins like CBL, GRB2 or CD19. Overall, SYK regulates anti-apoptotic mammalian target of rapamycin (mTOR), NF*κ*B, and STAT3 pathways [37, 38]. Overexpression of SYK has been reported in anaplastic lymphoma kinase positive tumors. Constitutive activation of SYK and BCR signaling is essential for cell proliferation and survival in different B-cell malignancies [39–41]. Inhibition of SYK has shown promising results in Hodgkin lymphoma and leukemia [41]. SYK expression was studied in HNSCC and 6 out of 10 cell lines were found to be SYK-positive. In addition, HNSCC samples from a Thai population showed higher SYK expression in lymph nodes as compared to HNSCC tumors and adjacent normal tissues. A significant correlation was found between expression of SYK and recurrence of the disease in HNSCC patients [42].

In our study, we observed SYK expression in all TE-series ESCC cell lines except TE-8. Expression of SYK was also not detectable in non-neoplastic esophageal epithelial cell line Het-1A. Recently, SYK overexpression has been reported in triple negative breast cancers and ovarian cancer. SYK has been suggested as a potential druggable target for a subset of triple negative breast cancers [43]. We evaluated the effect of inhibiting SYK in ESCC cell lines. SYK acts as a tumor suppressor in pancreatic adenocarcinoma and regulates cellular growth and invasion [44]. There is a great variation in the expression of SYK in epithelial cancers including breast and HNSCC [42].

Since SYK provides a pro-survival signal and it can modulate tumorigenesis via epithelial-mesenchymal transition, it could serve as a therapeutic target in cancers [45]. Indeed, SYK has been a target of interest in B cell acute lymphoblastic leukemia [46] and lymphomas [47]. SYK inhibitors including fostamatinib and BAY61-3606 have been reported to reduce growth of B-ALL *in vitro* [46]. Interestingly, a combination of PI3 kinase inhibitor (idelalisib, GS-1101) and SYK inhibitor (fostabatinib) reduced the survival of CLL-B cells and induced inhibition of leukemic B cell growth in a synergistic manner. This combination also disrupts chemokine signaling, a key for CLL microenvironment interaction [48–52]. The studies in prostate cancer reported that SYK supports migration and growth of prostate cancer cells [53]. SYK also plays an important role in ovarian cancer as paclitaxel-resistant cells overexpress SYK. The ratio of phosphorylated SYK vs SYK was found to be positively associated with paclitaxel- resistance in ovarian cancer cells *in vitro* [54]. Furthermore, paclitaxel resistant SYK overexpressing ovarian cancer was targeted in pre-clinical models to show that use of chemical inhibitor against SYK could be useful to decelerate tumor activity [55].

A number of phase II trials are ongoing in chronic lymphocytic leukemia (CLL) using entospletinib [56–58]. We investigated the effect of inhibiting SYK using entospletinib in ESCC cell line and a pre-clinical mouse model.

### siRNA based knockdown of SYK inhibits cell proliferation and cell invasion/migration in TE11 cells

**Figure 3:**
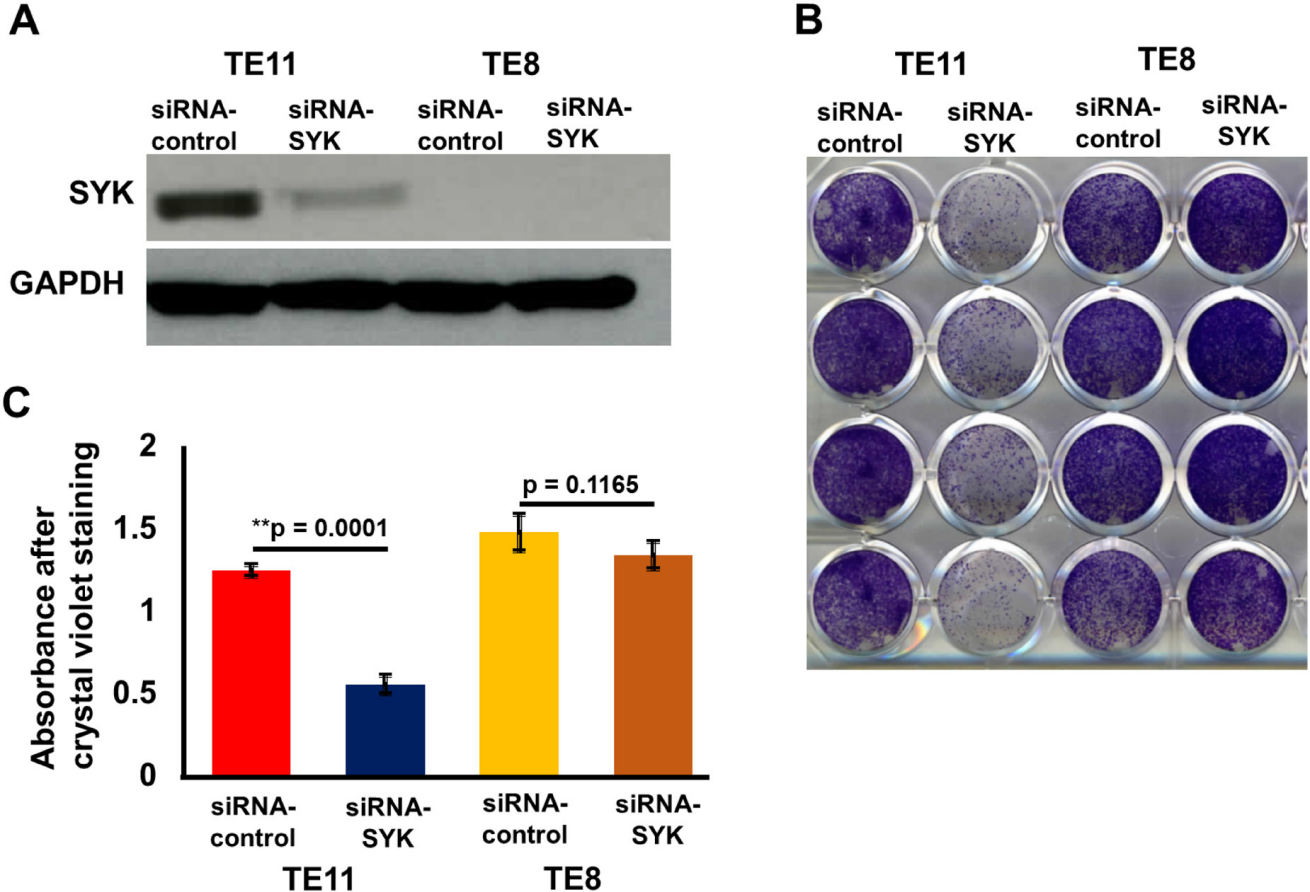
Effect of siRNA mediated knock down of SYK on cell proliferation, invasion/migration **(A)** Western blot showing siRNA mediated knockdown of SYK expression in TE11 and TE8 cell lines. **(B)** Cell proliferation assays in TE11 and TE8 cell lines after knockdown of SYK. **(C)** Histogram showing crystal violet absorbance in TE11 and TE8 cell lines post siRNA mediated knockdown of SYK.

**Figure 4:**
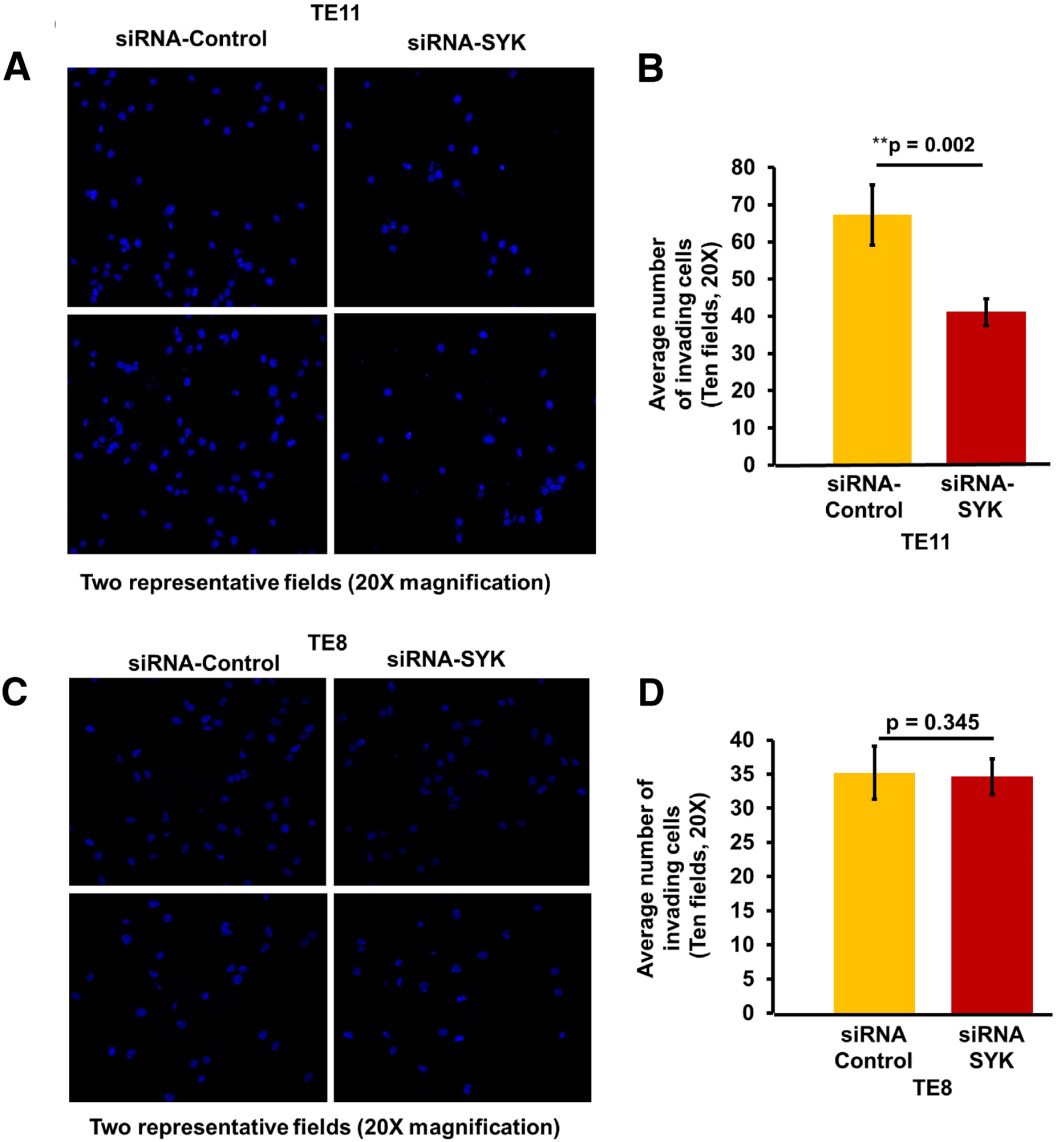
Effect of siRNA mediated knock down of SYK on invasion ability of TE11 and TE8 cell lines **(A)** Representative image to show number of TE11 cells that invaded matrigel with and without knockdown of SYK. **(B)** Histogram showing relative difference in invasion of TE11 cells with and without knockdown of SYK. **(C)** Representative image to show number of TE8 cells that invaded matrigel with and without knockdown of SYK. **(D)** Histogram showing relative difference in invasion of TE8 cells with and without knockdown of SYK

The siRNA transfection studies were carried out in both SYK positive (TE11) and negative ESCC cell lines (TE8) (Figure 3A). Proliferation of TE11 cells was significantly reduced compared to scrambled siRNA controls. No effect on proliferation was observed in TE8 (Figure 3B, 3C). siRNA based knockdown of SYK significantly decreased (p<0.002) invasion/migration capability of TE11 cells (Figure 4A, 4B). Similar data for inhibition of chemotaxis using siRNA based targeting of SYK has been observed in nasopharyngeal [59], endothelial [60] and aortic muscle cells [61]. We further evaluated efficacy of SYK selective inhibitor entospletinib that is already in phase II clinical trials for the treatment of hematological malignancies.

### SYK inhibition affects cell proliferation and growth rate in TE11 cells

**Figure 5:**
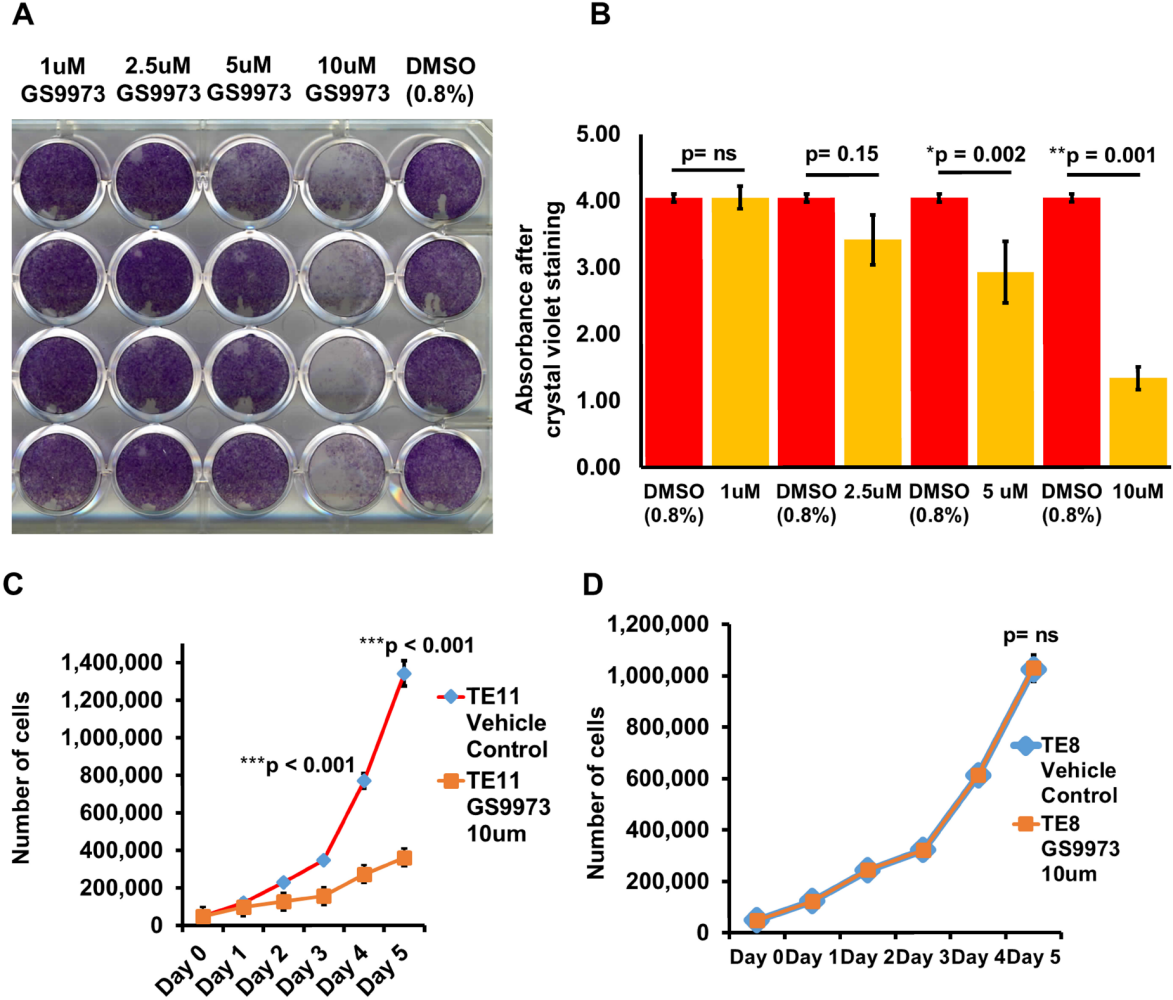
Effect of SYK inhibition using entospletinib/GS9973 on cell proliferation and cell doubling time of TE11 and TE8 cells **(A)** Image showing crystal violet stained TE11 cells treated with various concentrations of entospletinib/GS9973. **(B)** Histogram showing relative absorbance after crystal violet staining of TE11 cells treated with various concentrations of entospletinib/GS9973. **(C)** Growth rate of TE11 cell line treated with 10uM entospletinib/GS9973. **(D)** Growth rate of TE8 cell line treated with 10uM entospletinib/GS9973.

We treated TE11 (SYK positive) and TE8 (SYK negative) ESCC cell lines with 10 μM entospletinib or vehicle (Figure 5A, 5B) in a crystal violet assay. Inhibition of SYK significantly reduced proliferation of TE11 cells. In addition, we carried out cell doubling time assay with cell counting and observed the same effect of the drug in TE11 cell line (Figure 5C). However, the drug had no effect on TE8 cell line (Figure 5D).

### Entospletinib inhibits *in vivo* tumor growth in TE11 mouse xenografts

**Figure 6:**
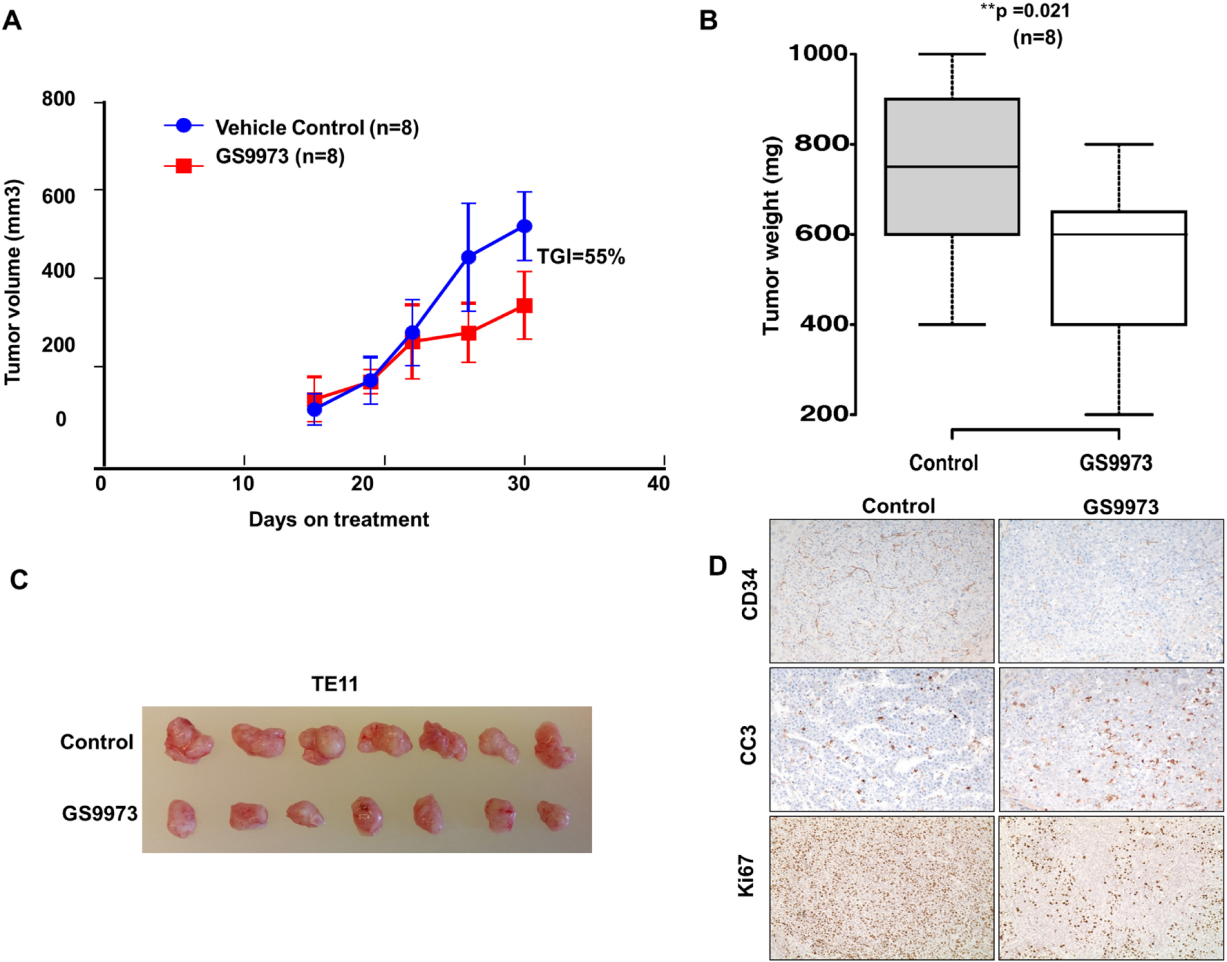
Effect of SYK inhibitor entospletinib/GS9973 on growth of subcutaneous xenograft of TE11 cell line **(A)** Tumor growth in mice untreated or treated with SYK inhibitor entospletinib. **(B)** Box plot showing tumor weight in treatment and control group. **(C)** Representative tumors from four mice untreated or treated with entospletinib/GS9973. **(D)** Representative image of immunohistochemistry using anti CD34, Ki67 and cleaved caspase 3 on control and treated tumor tissue sections.

We tested all ESCC cell lines for their ability to grow as xenograft tumors in mice for *in vivo* pre-clinical evaluation of entospletinib. TE11 successfully formed tumors in mice. We xenografted TE11 cell line in 8 mice and randomized into two groups of four each and started treating the mice with entospletinib or vehicle after 10 days of subcutaneous injections. We observed a significant reduction in tumor size in the treated group compared to the control with tumor growth inhibition of 55% (Figure 6A). We observed significant reduction in tumor weight after necropsy in treatment group compared to control (Figure 6B, 6C). The expression of cell proliferation marker Ki-67 was reduced in tumors treated with entospletinib while there was increased expression of cleaved caspase 3 showing trigger of programmed cell death in the grafted tumors (Figure 6D). Immunostaining with CD34, a marker of vascular endothelial progenitor cells, showed decreased expression upon treatment with entospletinib (Figure 6D). Overall, our data suggest that SYK can be targeted using selective inhibitors like entospletinib in ESCC and these findings can be extrapolated in cancers where SYK overexpression has been reported.

## DISCUSSION

ESCC is one of the devastating cancers with poor survival rates. Chemotherapy and radiation remains mainstay for treating advanced cases of ESCC. Currently, targeted therapies are not available for treating ESCC. Whole genome/exome sequencing studies have been recently carried out to identify genetic anomalies associated with ESCC. Genes with activating mutations that are recurrent among ESCC tumors could be potentially targeted for therapeutic intervention. Similarly, gene expression and protein expression profiling studies have also been carried out to identify differentially expressed genes that could be potentially targeted.

Reverse phase protein arrays (RPPA) offer an unbiased way of monitoring large number of proteins from biospecimens. We employed this high-throughput methodology to monitor expression of 272 proteins across seven ESCC cell lines and a non- neoplastic esophageal epithelial cell line. The details of the antibodies as provided in the RPPA Array Core website of MD Anderson Cancer Centre is provided in Supplementary Table 3. We identified several proteins that were differentially expressed in ESCC cell lines compared to non-neoplastic esophageal epithelial cell line. Spleen associated tyrosine kinase (SYK) was found to be overexpressed in six out of seven cell lines that were investigated. Functional studies in esophageal cancer cell line showed siRNA mediated knock down of SYK inhibited proliferation, invasion/migration capability of cells. In addition, pharmacological inhibition of SYK in cell lines and mouse xenografts showed similar effects. Considering lack of effective targeted therapeutic agents to treat ESCC, these preclinical studies might prove valuable in prioritizing reliable candidates. SYK warrants further studies to determine its clinical utility as a potential target to treat ESCC. Our study provides a proof principle for further clinical investigations about the therapeutic potential of entospletinib in ESCC as the drug is already in clinical trials for other cancers.

## MATERIALS AND METHODS

### Cell lines and cell culture

Esophageal squamous cell carcinoma cell lines TE1, TE2, TE5, TE8, TE10, TE11 and TE15 were grown in DMEM high glucose with 10% FBS supplement. The cell lines were kind gift from Dr. Anil K Rustgi, Division of Gastroenterology, University of Pennsylvania, USA. Non-neoplastic esophageal epithelial cell line Het-1A (purchased from ATCC) was grown in bronchial epithelial cell basal medium (BEBM) along with the required additives obtained from Lonza/Clonetics Corporation as a kit along with the media (Media Kit Catalog No. CC-3170). Cell lines were serum starved for 8 hours, washed in PBS three times and harvested in lysis buffer as described below. The details of ESCC and normal cell lines are provided in Table 1. These cell lines were authenticated by short tandem repeat profiling at the Johns Hopkins Genetic Research Core Facility.

**Table 1:** Details of esophageal cancer cell lines and non-neoplastic esophageal epithelial cell line used in the study

	Name of Cell line	Differentiation characteristics of the cell lines
**1**	TE1	Well
**2**	TE2	Poor
**3**	TE5	Poor
**4**	TE8	Moderate
**5**	TE10	Well
**6**	TE11	Moderate
**7**	TE15	Well
**8**	HET-1A	Normal esophageal squamous cells

### Reverse phase protein array (RPPA)

Proteins were extracted from all the cells in Lysis buffer (1% Triton X-100, 50mM HEPES, pH 7.4, 150mM NaCl, 1.5mM MgCl2, 1mM EGTA, 100mM NaF, 10mM Na pyrophosphate, 1mM Na3VO4, 10% glycerol, protease and phosphatase inhibitors from Roche Applied Science Cat. 05056489001 and 04906837001). Protein quantity was measured using BCA protein assay and normalized in SDS-PAGE. Proteins from each cell line was mixed with 4X SDS sample buffer (40% Glycerol, 8% SDS, 0.25M Tris- HCL, pH 6.8) to a final concentration of 1X sample buffer and 1.5 ug/ul protein concentration. The lysates were boiled for five minutes and 40ul of the same were sent to RPPA core facility of MD Anderson Cancer Centre for further analysis in technical replicates. Remaining samples were frozen for future use in validation.

### Data acquisition and analysis

All the samples were analyzed following published protocols [62]. In brief, cell lysates were two-fold-serial diluted for 5 dilutions (from undiluted to 1:16 dilution) and arrayed on nitrocellulose-coated slide in 11×11 format. Samples were probed with 272 antibodies by CSA amplification approach and visualized by DAB colorimetric reaction. The slides were scanned in flatbed scanner and the spots were quantified in Array Pro Analyzer. Relative protein levels for each sample were determined by interpolation of each dilution curves from the “standard curve” (supercurve) of the slide (antibody). The construction of supercurve was done using a script in R-environment [63]. These values (given as Log2 values) are defined as Supercurve Log2 (Raw) value and shown in the page of “RawLog2”. All the data points were normalized for protein loading and transformed to linear value, designated as “Normalized Linear” value. The linear value was used to compare differentially expressed proteins in ESCC cell lines compared to normal cell line (Supplementary Table 2). A detailed procedure is provided in Supplementary Method.

### Western blotting

Cells from the procedure above were pelleted by centrifugation, washed twice with PBS, and lysed with modified RIPA buffer (150 mM NaCl, 50 mM Tris⋅HCl pH 7.4, 1% NP-40, 0.25% sodium deoxycholate, 1 mM EDTA, 1 mM sodium orthovanadate, 10 mM NaF, 10 mM glycerophosphate, 5 mM sodium pyrophosphate) containing 1% of human protease inhibitor cocktail (Sigma Aldrich) at 4°C. The protein content of the whole cell lysates was quantified using a detergent compatible (DC) protein assay (Bio- Rad). Lysates in sample buffer comprised of 720 mM 2-mercaptoethanol, 0.001% bromophenol blue, 2% SDS, 10% glycerol, 80 mM Tris⋅HCl pH 6.8 were denatured at 95°C for 5 min. A sample containing 30 μg of total protein was resolved on 4-20% Criterion Precast Gel (Bio-Rad) SDS-PAGE, followed by transfer using polyvinylidene difluoride (PVDF) membrane (Millipore). After blocking with 5% bovine serum albumin (BSA) in TBST (20 mM Tris⋅HCl, 137 mM NaCl, 0.1% Tween-20 pH 7.6) for one hour at room temperature, the membrane was incubated with primary antibody overnight at 4°C. The primary antibodies included a mouse anti-SYK antibody (clone 4D10, catalog # SC-1240, Santa Cruz Biotechnology), and a rabbit anti-GAPDH (clone 14C10, Catalog # 2118, Cell Signaling Technology). All primary antibodies were used at 1:1000 dilution in TBST containing 5% BSA. After washing three times with 10 ml of TBST, the membranes were incubated with HRP-labeled anti-rabbit (sc-2030, Santa Cruz Biotechnology) or HRP-labeled anti-mouse (sc-2031, Santa Cruz Biotechnology) secondary antibodies with a dilution of 1:5000 TBST containing 5% BSA for one hour at room temperature. After washing twice with TBST, the membranes were incubated with horseradish peroxidase (HRP)-labeled anti-rabbit (sc-2030, Santa Cruz Biotechnology) or HRP-labeled anti-mouse (sc-2031, Santa Cruz Biotechnology) secondary antibodies with a dilution of 1:5000 for 1 h at room temperature. Protein- antibody complex signals were detected by exposing the X-ray films after treatment with enhanced chemiluminescence (ECL) kit (Pierce Thermo Scientific).

### siRNA transfection of TE11 and TE8 cells

TE11 and TE cell lines were transiently transfected using siRNA-*SYK* and siRNA scrambled control-using lipofectamine RNAiMax by following the manufacturer's protocol (Catalog # 13778075, ThermoFisher). We performed siRNA-based knockdown of SYK in TE11 and TE8 cell lines. The expression of SYK was transiently knocked down using 25pmol of ON-TARGETplus Human SYK smart pool siRNA (Catalog # L- 003176-00-0010; Dharmacon). Allstars negative control siRNA was purchased from Qiagen (Catalog # 1027281), it was used for siRNA-scrambled control transfection at a final concentration of 25 pM. The day prior to transfection, cells were seeded at 60% confluence in six well plates and siRNA transfection was carried out next day, the knockdown of the target was checked by western blot after 36 hours post-transfection.

### Cell proliferation and crystal violet staining

Cells were trypsinized after 36 hours of siRNA transfection and 5000 cells were seeded onto four wells of 24 well plates in triplicate and stained with 0.5% crystal violet. Cells were then fixed with 0.1% acetic acid for 5 min and stained with 0.5% crystal violet dye (in methanol:deionized water, 1:5) for 10 min. The excess crystal violet dye was removed by five washes in deionized water on a shaker (each was for 10 min) and the culture plates were dried overnight. The crystal violet dye was released from cells by incubation with 1% sodium dodecyl sulfate (SDS) for 6 h before optical density (OD)595 nm measurement. Similarly, cells were seeded at a density of 5000 cells per well in 24 well plates and treated with entospletinib at different concentrations (1 μM, 2.5μM, 5μM and 10μM) for five days. Crystal violet assays were performed to determine the effective dose of entospletinib on both cell lines for cell doubling time assay.

### Cell doubling time assay

A total of 5 × 104 cells (TE11 and TE8) were seeded in duplicate in a six well plate. entospletinib (10 μM) treatment and vehicle control (0.8% DMSO) were started from Day 0 (next day of plating) and continued till Day 5. The cells were counted using Z™ Series COULTER COUNTER^®^ for next six days with or without SYK inhibitor treatment.

### Invasion/migration assays and imaging

Post 36 hours of siRNA transfection, cells were trypsinized and 5×104 cells seeded onto Biocoat matrigel invasion chambers (catalog # 354480, BD Biosciences) in high glucose DMEM containing 2% FBS. DMEM high glucose medium with 10% FBS was added in the lower chamber as chemo attractant. The cells were allowed to invade the matrigel membrane overnight. The membranes were stained with DAPI (Invitrogen) after carefully removing the cells from the inner side and the number of cells that had invaded the matrigel and membrane was counted in 10 randomly selected fields at 20X magnification under fluorescence microscope using blue/cyan filter. Cells were counted using ImageJ and average number of cells in siRNA- control and siRNA-*SYK* in TE11 and TE8 lines were calculated separately to evaluate the differences in invasion between siRNA-control and siRNA-*SYK* transfected TE11 and TE8 cells.

### RT-PCR analyses in ESCC and normal esophageal cell lines

A total of 5 × 106 cells were taken from TE8, TE-11 and Het-1A cell lines. Total RNA was isolated from all the cell lines and cDNA library was prepared. PCR reaction was performed in 20 μl reaction volume as described previously. PCR conditions - 94°C for 3 min; 35 cycles of 94°C for 30s, 55-60°C (annealing temperature: 56°C for *GAPDH*, 53°C for SYK, for 30 s, and 72°C for 1 min; followed by 72°C for 5 min. PCR products were separated on a 2% agarose gel and stained with ethidium bromide. Details of the primers used for RT-PCR are provided in Table 2.

**Table 2:** List of primers used for RT-PCR assay

RT-PCR primers		
Primer	5’ Sequence 3’	Amplicon Size (Bps)
*GAPDH*-FP	TGGTCACCAGGGCTGCTT	151
*GAPDH*-RP	AGCTTCCCGTTCTCAGCCTT	
*SYK*-FP	CATGTCAAGGATAAGAACATCATAGA	514
*SYK*-RP	AGTTCACCACGTCATAGTAGTAATT	

FP: forward primer; RP: reverse primer.

### Mouse xenograft studies with SYK inhibitor entospletinib

All the procedures involved in animal work was approved by the JHMI institutional animal care and use committee and were performed in accordance with the IACUC. All the seven cancer cell lines were tested for their ability to graft tumors in NSG mice (NOD.Cg-*Prkdcscid Il2rgtm1Wjl*/SzJ). The characteristics of these mice combine the features of the NOD/ShiLtJ background, the severe combined immune deficiency mutation (*SCID*) and IL2 receptor gamma chain deficiency that results in lack of mature T, B or functional NK cells, and are deficient in cytokine signaling in NSG mice leading to better engraftment of cells of interest. Only TE11 cell line formed tumors after subcutaneous injection of 2 million cells per site on both flanks of mice. To evaluate the therapeutic potential of targeting SYK with entospletinib, TE11 cells were injected subcutaneously into flanks of NOD-SCID mice. Tumor growth was measured twice a week and the tumor volume was estimated as [Tumor Volume=(BreadthxBreadth)xLength/2]. Once the tumors attained desired volume (~80mm3), animals were segregated into treatment (n=4) and control (n=4) groups. Each group was treated using subcutaneous injection of entospletinib at 10-mg/Kg-body weight thrice a week. The animals were followed for 30 days and tumor volumes were recorded. Body weight was measured during treatment process to see if treatment induced toxicity leads to weight loss in animals.

### Immunohistochemistry

The xenograft tumors were fixed in 10% buffered formalin (Formalin, Buffered, 10% (Phosphate Buffer/Certified), Fisher Chemical, USA) and paraffin blocks and tissue sections were prepared using standard protocols. Immunohistochemistry on the subcutaneous xenograft tumor sections were carried out using an anti-CD34 antibody from Abcam (ab8536, mouse monoclonal antibody) at a dilution of 1:200. Rabbit monoclonal antibody against cleaved caspase-3 ((Asp175) (D3E9) CST #9579) from Cell Signalling Technology was used at 1:200 dilution for immunostaining. Rabbit monoclonal anti- Ki-67 Antibody (MA5-14520) from ThermoFisher Scientific, USA was used to immunostain proliferating cells. The Poly-HRP anti-Rabbit IHC Detection Systems (Leica Biosystems, USA) and DAB chromogen system (Vector Labs, USA) were used for detection of signals. Counterstaining was carried out with Hematoxylin QS (H-3404, Vector Labs, USA) and quenched in Scotts buffer (Catalogue, 3802900, Leica Biosystems, USA).

### Statistical analysis

Statistical analysis was done using GraphPad Prism software (v. 5.0c; San Diego, CA). Statistical significance was determined by using paired or unpaired Student's t test or one-way ANOVA followed by Bonferroni correction's multiple comparisons test. Statistical differences for the mean values are indicated as follows: ^*^, *p* < 0.05; ^**^, *p* < 0.01; ^***^, *p* < 0.001; and ^****^, *p* < 0.0001. Unless indicated, data are presented as the mean ± SEM.

## SUPPLEMENTARY MATERIALS FIGURES AND TABLES









## Abbreviations

BEBM: bronchial epithelial cell basal medium
EAD: esophageal adenocarcinoma
ECL: enhanced chemiluminescence
ESCC: esophageal squamous cell carcinoma
HCC: hepatocellular carcinoma
HNSCC: head and neck squamous cell carcinoma
HRP: horseradish peroxidase
iTRAQ: isobaric tags for relative and absolute quantitation
MRM: multiple reaction monitoring
OD: optical density
PTMs: post-translational modifications; PTM
RPPA: reverse phase protein array
TMT: Tandem mass tags
SILAC: stable isotope labeling with amino acids in cell culture
SDS: sodium dodecyl sulfate
SYK: spleen tyrosine kinase.
