# Supplementary material for: Identification of spleen tyrosine kinase as a potential therapeutic target for esophageal squamous cell carcinoma using reverse phase protein arrays
Source: Oncotarget. 2018 Jan 1;9(26):18422–34. doi: 10.18632/oncotarget.24853 (PMC5915082; doi:10.18632/oncotarget.24853)
Supplement: Supplementary file 1 [file oncotarget-09-18422-s001.pdf]

Identification of spleen tyrosine kinase as a potential therapeutic target for esophageal squamous cell carcinoma using reverse phase protein arrays

SUPPLEMENTARY MATERIALS

Details of procedure employed for Reverse Phase Protein Array (RPPA core facility MD Anderson Cancer Centre)
1) Tumor or cell lysates were two-fold-serial diluted for 5 dilutions (from undiluted to 1:16 dilution) and arrayed on nitrocellulose-coated slide in 11x11 format.
2) Samples were probed with antibodies by CSA amplification approach and visualized by DAB colorimetric reaction.
3) Slides were scanned on a flatbed scanner to produce 16-bit tiff image.
4) Spots from tiff images were identified and the density was quantified by Array-Pro Analyzer.
5) Relative protein levels for each sample were determined by interpolation of each dilution curves from the “standard curve” (supercurve) of the slide (antibody). Supercurve is constructed by a script in R written by Bioinformatics. These values (given as Log2 values) are defined as Supercurve Log2 (Raw) value and shown in the page of “RawLog2”.
6) All the data points were normalized for protein loading and transformed to linear value, designated as “Normalized Linear” (Labeled “NormLinear” in worksheet).
*The linear value can be used for “bar graph” or further analysis according to your study design.
We have recently implemented an improved normalization algorithm for protein loading correction and antibody variation adjustment. This approach is critical to provide accurate values for RPPA data merging if you have samples performed in separate RPPA sets that you wish to combine together. We listed the protein loading correction factor (CF) in the last column on this page (NormLinear) for your reference. Each sample has its unique correction factor. If the correction factor is less than 0.25 or greater than 2.5, we consider these samples “outliers,” indicating that protein concentration is much lower or much higher than the other samples. We suggest that you exclude these “outliers” from further analysis. (One exception: intrinsic protein expression patterns can skew the correction factor to some extent. In that case, you may want to include this data, but we suggest that you examine the data set carefully.)
7) “Normalized Linear” value was transformed to Log2 value (Labeled “NormLog2” in worksheet), and then median-centered for Hierarchical Cluster analysis (Labeled “NormLog2_MedianCentered” in worksheet). Median-Centered values were then formatted for heatmap generation in the “Format for Heatmap” worksheet.
8) We included heatmaps for an Unsupervised Hierarchical cluster (unsupervised on both antibodies and samples) and an antibody unsupervised but samples are arranged in the order you submitted them, for your reference.
9) The heatmap included was generated in Cluster 3.0 (http://www.eisenlab.org/eisen/) as a hierarchical cluster using Pearson Correlation and a center metric. The resulting heatmap was visualized in Treeview {http://www.eisenlab.org/eisen/) and presented as a high resolution.bmp format.
10) We stained 272 unique antibodies and 4 types of secondary antibody negative controls which were analyzed on Array-Pro then by supercurve Rx64 3.1.1. There were 20 sets of replicated antibodies among 272 antibodies. We performed QC test for each antibody staining (slide). The QC Score (Probability) values were also included in the data spreadsheet for your references. QC score above 0.8 indicates good antibody staining. We only included in the data for the 272 individual antibodies with QC Scores higher than 0.80 in the Heatmaps. In the case of antibodies with replicates, the one with the highest QC Score was usually used. There are 51 individual mouse antibodies (labeled “-M”) that will be removed for mouse tissue or xenograft samples, unless you specifically indicated for inclusion in your sample submission form. There are 217 Ab reported for xenograft samples.
11) The bioinformatics should be done at your end from the Excel file we provide. We recommend that you create bar graphs based on the data in the Excel file. The heatmap is solely to provide overall parterns.

Antibody status for RPPA
(V) = Validated antibody for RPPA
(C) = Use with caution. Validation in progress
(QC) = These antibodies recognize unidentifued “damaged” component(s) in addition to its specific protein. The “damaged” component(s) were observed only in certain tissue samples.
(E) = Under Evaluation
(M) = Mouse antibody was used.
(G) = goat antibody was used.
(R) = Rabbit Antibody was used.
(T)= Rat Antibody was used.

** ***Example: Akt_pS473-R-V_GBL9016996 means antibody specifically recognizes Akt phosphorylated on Serine 473. This is a rabbit antibody validated for RPPA application. GBL9016996 is the slide ID (barcode). The slide ID is not included in the final version of heatmap***.

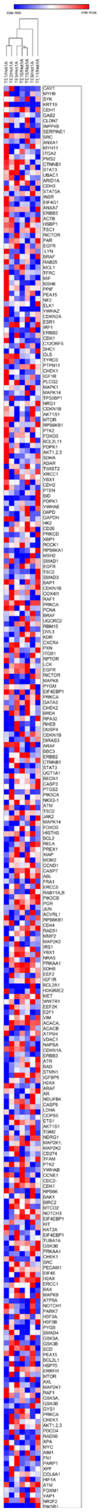

Supplementary Figure 1: Heatmap of differentially expressed proteins in ESCC cell lines with unsupervised hierarchical clustering.

Supplementary Table 1: Relative difference in protein expression levels in ESCC cell lines compared to Het-1A

See Supplementary File 1

Supplementary Table 2: Raw and processed RPPA data from the study

See Supplementary File 2

Supplementary Table 3: Details of antibodies used in RPPA array

See Supplementary File 3
